# Deep molecular response in patients with chronic phase chronic myeloid leukemia treated with the plasminogen activator inhibitor‐1 inhibitor TM5614 combined with a tyrosine kinase inhibitor

**DOI:** 10.1002/cam4.5292

**Published:** 2022-09-23

**Authors:** Naoto Takahashi, Yoshihiro Kameoka, Makoto Onizuka, Yasushi Onishi, Fumiaki Takahashi, Takashi Dan, Toshio Miyata, Kiyoshi Ando, Hideo Harigae

**Affiliations:** ^1^ Akita University School of Medicine Akita Japan; ^2^ Tokai University School of Medicine Isehara Japan; ^3^ Tohoku University, Sendai Japan; ^4^ Iwate Medical University Morioka Japan; ^5^ Renascience Inc. Tokyo Japan

**Keywords:** *BCR‐ABL1*, chronic myeloid leukemia, PAI‐1 inhibitor, tyrosine kinase inhibitor

## Abstract

**Background:**

We recently showed that pharmacological inhibition of plasminogen activator inhibitor‐1 (PAI‐1) activity, based on TM5614, increases cell motility and induces the detachment of hematopoietic stem cells from their niches. In this TM5614 phase II clinical trial, we investigated whether the combination of a PAI‐1 inhibitor and tyrosine kinase inhibitors (TKIs) would induce a deep molecular response (DMR) in patients affected by chronic myeloid leukemia (CML) by quantifying *BCR‐ABL1* transcripts.

**Methods:**

Patients with chronic phase CML treated with a stable daily dose of TKIs for at least 1 year and yielding a major molecular response (MMR) but not achieving MR^4.5^ were eligible for this study. After inclusion, patients began to receive TM5614 as well as a TKI. The primary objective was an evaluation of the cumulative incidence of patient progression from an MMR/MR^4^ to MR^4.5^ by 12 months.

**Results:**

Thirty‐three patients were enrolled in the study. The median age was 59.0 years and 58% were male. No Sokal high‐risk patients were enrolled in this trial. The median TKI treatment duration was 4.8 years. At the start of this study, seven patients and 26 patients received imatinib and second‐generation TKIs, respectively. The cumulative MR^4.5^ incidence by 12 months was 33.3% (95% confidence interval, 18.0%–51.8%). The cumulative MR^4.5^ spontaneous conversion over 12 months was estimated as 8% with TKIs alone based on historical controls. The halving time of *BCR‐ABL1* at 2 months was significantly shorter for patients who achieved an MR^4.5^, by 12 months than for the other patients (cutoff value: 48 days; sensitivity: 0.80; specificity: 0.91; ROC‐AUC: 0.83). During this study, bleeding events and abnormal coagulation related to the drug were not reported, and TM5614 was found to be highly safe.

**Conclusion:**

TM5614 combined with TKI was well tolerated and induced MR^4.5^ in more patients than stand‐alone TKI treatment.

## 1 INTRODUCTION

Tyrosine kinase inhibitors (TKIs), which inhibit the function of *BCR‐ABL1* tyrosine kinase, have dramatically improved life expectancy for patients affected by chronic myeloid leukemia (CML).^1^ TKIs are the standard first‐line treatment for patients with CML.^2^, ^3^, ^4^ However, long‐term, continuous TKI treatments lead to severe adverse events, especially vascular occlusive disease, which affect patient quality of life and increase financial burdens.

In 2010, the prospective treatment‐free remission (TFR) study and the Stop Imatinib 1 (STIM1) trial presented results indicating that some patients with CML, who maintained a deep molecular response (DMR), could safely discontinue TKI treatments, and long‐term follow‐up confirmed that these patients maintained remission.^5^, ^6^ Following these pivotal studies, multiple TFR trials were conducted and demonstrated that some CML patients achieving a stable DMR could safely cease therapy without relapsing.^7^, ^8^, ^9^, ^10^, ^11^, ^12^, ^13^ Although DMR is the milestone of TFR according to these trials, newly diagnosed chronic phase CML can rarely achieve DMR because TKIs cannot eradicate CML stem cells that are quiescent in the bone marrow (BM) niche and are resistant to TKIs.^14^, ^15^, ^16^, ^17^

Recently, we developed a low‐molecular‐weight synthetic plasminogen activator inhibitor‐1 (PAI‐1) inhibitor.^18^, ^19^ In a mouse CML model, treatment comprising a combination of imatinib and a PAI‐1 inhibitor, TM5614, substantially improved therapeutic outcomes of the TKI, demonstrated by fewer CML cells in the BM and extended survival. Furthermore, the pharmacological inhibition of PAI‐1 combined with TKIs effectively eliminated leukemic stem cells (CML‐LSCs) in the BM.^20^ In this TM5614 phase II clinical trial, we investigated whether the PAI‐1 inhibitor combined with TKIs would induce a DMR in patients with CML, using the *BCR‐ABL1* transcript as a quantitative marker.

## 2 METHODS

### 2.1 Patients and synopsis of study protocol

This study was a prospective, multicenter, open‐label, single‐arm phase II trial (jRCT2031190071) conducted in accordance with the Declaration of Helsinki and Good Clinical Practice, which was approved by the Ethics Committee of Tohoku University (No. 2019–193,006) and by the ethics committees of all participating institutions. Written informed consent was obtained from all the participants before enrollment. Adult Ph chromosome‐positive patients with CML were eligible if they were (i) in the chronic phase, (ii) treated with TKIs for over 1 year without a dose modification within the last 12 weeks, and (iii) classified as having a major molecular response (MMR), defined by *BCR‐ABL1* ^*IS*^ ≤ 0.1% without MR^4.5^ defined by *BCR‐ABL1* ^*IS*^ ≤0.0032% at study initiation. The planned therapy consisted of a continuation of the same TKI at the same daily dose with the addition of TM5614 at 150–180 mg/day for 48 weeks.

### 2.2 Study endpoints and assessments

The primary endpoint comprised the percentage of patients achieving MR^4.5^ by 12 months, defined as a 4.5‐log reduction in the *BCR‐ABL1* transcript (*BCR‐ABL1* ^*IS*^ ≤0.0032% or undetectable disease in cDNA with >100,000 *ABL* transcripts) quantified by polymerase chain reaction (PCR) testing every 4 weeks during the study and performed according to the European Leukemia Net recommendations in a central laboratory (SRL Hachioji Laboratory, Japan). Secondary endpoints included safety and pharmacological analyses of TM5614 and *BCR‐ABL1* ^*IS*^ kinetics, including the half‐life (halving time) of *BCR‐ABL1* ^*IS*^. We cataloged adverse events based on the Common Terminology Criteria for Adverse Events version 4.03.

### 2.3 Statistical analyses

The planned sample size was calculated using Yate's chi‐square test and by estimating the minimum number of patients required to reject the null hypothesis with respect to the primary endpoint (≤8% MR^4.5^ rate by 12 months), which was estimated by the ENESTnd^21^, ^22^ and DASISION trials^23^ as a historical control. At minimum, 31 patients needed to be enrolled to achieve 80% power to reject the null hypothesis with a one‐sided α‐level of 5% if the true rate of MR^4.5^ by 12 months was ≥33%. With an actual patient enrollment of 33, the power increased to 82.7%, with all other assumptions remaining identical.

The primary endpoint was given as a percentage with 95% confidence interval (CI). To identify the covariates that predicted MR^4.5^, univariate and multivariate Cox regression analyses were conducted. The following variables were considered: patient age, sex, time since diagnosis, and *BCR‐ABL* ^*IS*^ value (%) at the baseline of the study. Further, we used a stepwise multivariate approach to ascertain the most important prognostic factors with a two‐sided variable retention criterion of *p* < 0.05. Variables with associations of marginal significance (two‐sided *p* < 0.15) were further used to build the multivariate model. Statistical analyses were conducted using EZR, a graphical user interface for R (version 2.13.0; The R Foundation for Statistical Computing.) and Stata (version 13.0; Stata Corporation). The data presented in this study were based on a cutoff date of December 17, 2020; at this time all patients enrolled in the study had either completed ≥48 weeks of therapy or discontinued the study.

## 3 RESULTS

### 3.1 Patient characteristics

From September 2019 to December 2019, 33 patients with CML, who were all in the chronic phase, were enrolled and screened. All eligible patients were evaluated and classified based on their age, sex ratio, height, body weight, prior interferon‐alpha (IFN‐α) treatment, Sokal risk score,^24^ time since diagnosis, and *BCR‐ABL* ^*IS*^ value (%) at the baseline of the study (Table 1). All patients were on frontline TKI treatment except one who had prior IFN‐α therapy.

**TABLE 1:** TM5614 phase II trial patient characteristics

Number of patients	33
Median age, years (min‐max)	59 (23–79)
Chronic CML phase (%)	100
Sex ratio (M/F)	19/14
Median height, cm (min‐max)	165.0 (147.5–184.3)
Median body weight, kg (min‐max)	59.7 (47.3–87.0)
ECOG Performance status (0/1/2/3‐)	32/0/1/0
Sokal score risk (Low/Intermediate/High/NA)	16/5/0/12
Median time since diagnosis, months (range)	58 (12–260)
<36 months	7
36 months – <60 months	10
60 months – <120 months	8
120 months	8
prior IFN‐α treatment (number)	1
prior TKI treatment (IM/DAS/NIL/BOS) [IM/2GTKI]	7/7/13/6 7/26
Median *BCR‐ABL* ^*IS*^ value, % (range) [MMR (≥0.01)/MR^4^ (<0.01)]	0.0113 (0.0036–0.0748) 17/16

Abbreviations: 2G, second‐generation; BOS, bosutinib; DAS, dasatinib; ECOG, Eastern cooperative oncology group; IFN‐α, interferon‐α; IM, imatinib; NIL, nilotinib; TKI, tyrosine kinase inhibitor.

### 3.2 TM5614 administration and pharmacokinetics

All patients started daily TM5614 at a dose of 150 mg/day, and this was increased to 180 mg/day after 8 weeks for 18 patients (54.5%) because those patients did not report any adverse events (AEs) or abnormal laboratory results and showed drug tolerance. One patient treated with nilotinib discontinued the study after 6 months because of cardiac catheterization due to coronary artery disease, a complication that was not related to TM5614. The plasma concentrations of TM5614 were measured in all patients at 4, 12, 24, and 48 weeks. The median value was 12,300 ng/ml (range, 4040–58,600 ng/ml), which changed within a certain range in each patient, and no accumulation of TM5614 was observed after 48 weeks.

### 3.3 Molecular efficacy of the combination of TM5614 and TKIs

**FIGURE 1:**
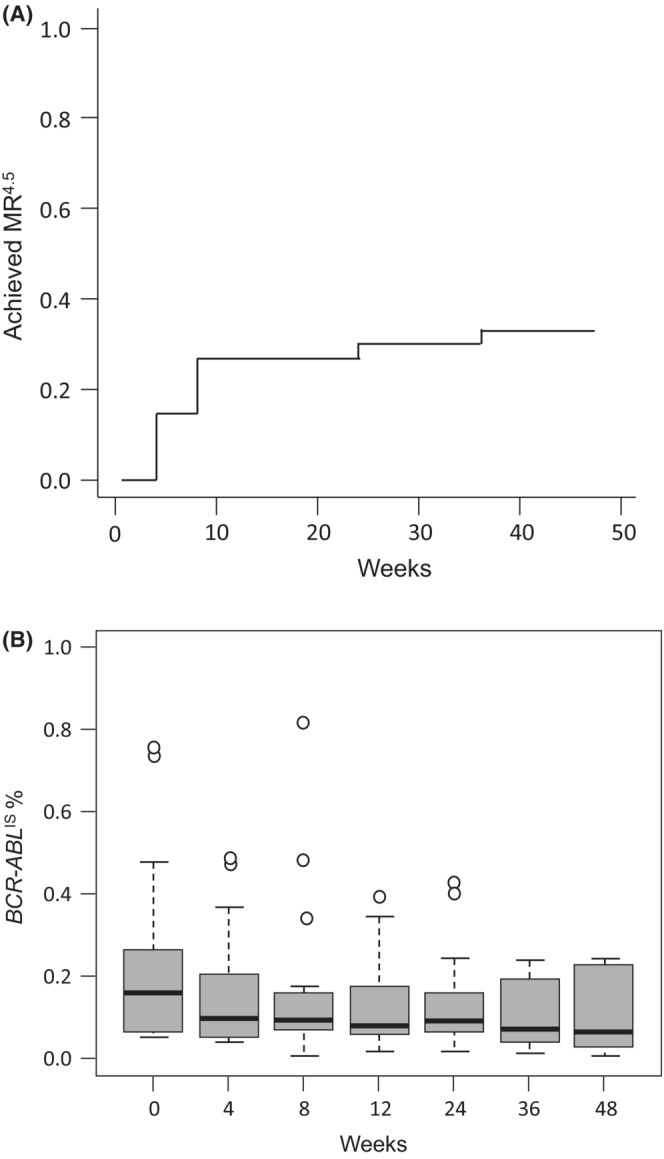
Molecular efficacy of the TM5614 combination. (A) Cumulative incidences of a MR^4.5^ conversion by 48 weeks. Eleven patients (33.3% [95% confidence interval (CI), 18.0%–51.8%]) achieved MR^4.5^ during 48 weeks. The median time to MR^4.5^ was 8 weeks [95% CI, 4–24]. (B) *BCR‐ABL* ^*IS*^ % kinetics within 48 weeks. Boxes, 5–95 percentiles; horizontal bars, median; vertical brackets, ranges.

Eleven patients achieved MR^4.5^ within 48 weeks. The cumulative incidence of MR^4.5^ was 33.3% [95% CI, 18.0%–51.8%] by 48 weeks, exceeding the predefined threshold success rate of 33% (Figure 1A). The median time to MR^4.5^ was 8 weeks [95% CI, 4–24 weeks]. *BCR‐ABL* ^*IS*^ % kinetics are shown using a boxplot in Figure 1B.

### 3.4 Log‐reduction in *BCR‐ABL* ^*IS*^ % during this study

**FIGURE 2:**
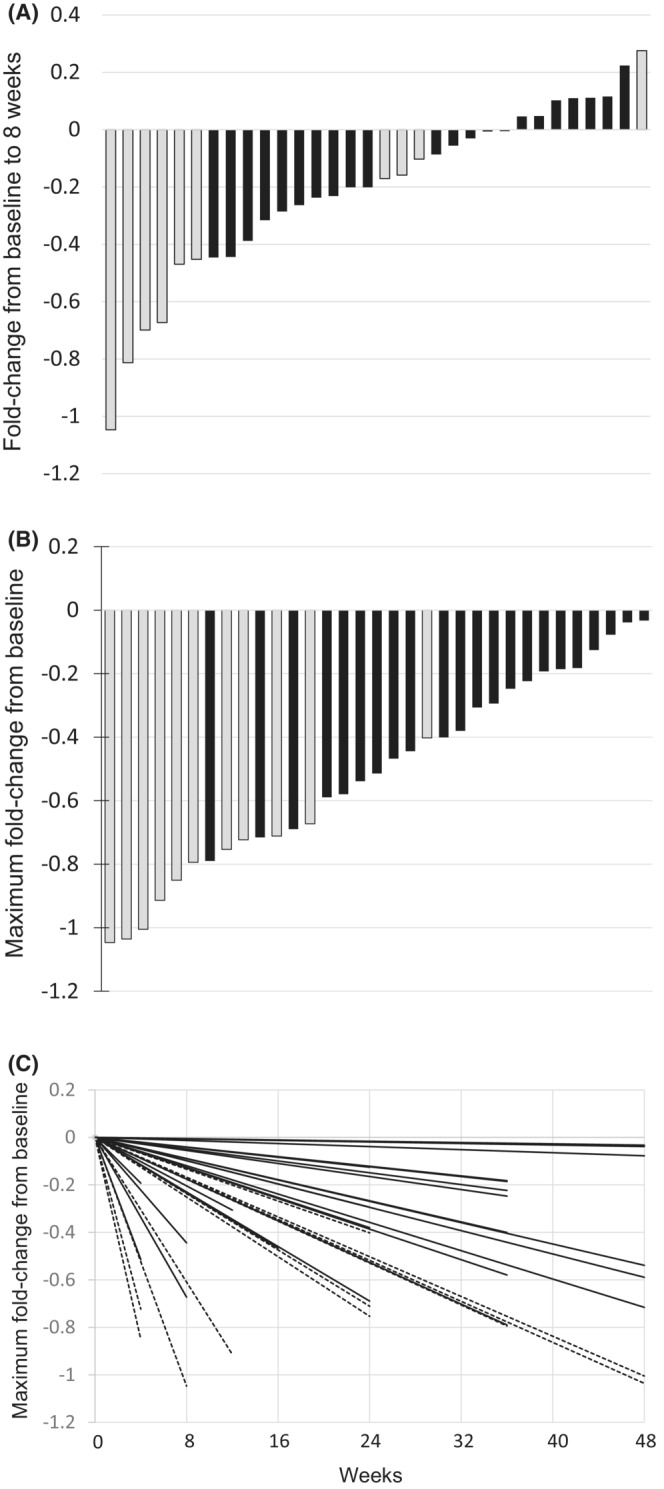
Log‐reduction in *BCR‐ABL* ^*IS*^ % during this study. (A) Waterfall plot indicating the log‐change in *BCR‐ABL* ^*IS*^ % at 8 weeks from the baseline. (B) Waterfall plot indicating the log‐reduction in the best response from the baseline per patient. Gray or black bar indicating whether or not, respectively, a MR^4.5^ was achieved. **C.** Spider plot indicating the log‐reduction in the best response from baseline in *BCR‐ABL* ^*IS*^ % per patient during TM5614 administration. The dashed line or black line indicates whether or not a MR^4.5^ was achieved, respectively.

The log‐reduction in *BCR‐ABL* ^*IS*^ % was calculated from the values at baseline to 8 weeks or the time of the best response during this study. The *BCR‐ABL* ^*IS*^ % changes were shown using a waterfall plot in Figure 2A,B. All patients showed a log‐reduction of *BCR‐ABL* ^*IS*^ % during the study, and patients with a larger reduction at 8 weeks tended to achieve a MR^4.5^. A spider plot showed the log‐reduction of *BCR‐ABL* ^*IS*^ % from the baseline to the time of best response during this study. Patients who achieved MR^4.5^ in the study showed more rapid reduction than those without, as shown in Figure 2C.

### 3.5 Halving time of *BCR‐ABL* ^*IS*^ % at 8 weeks

**FIGURE 3:**
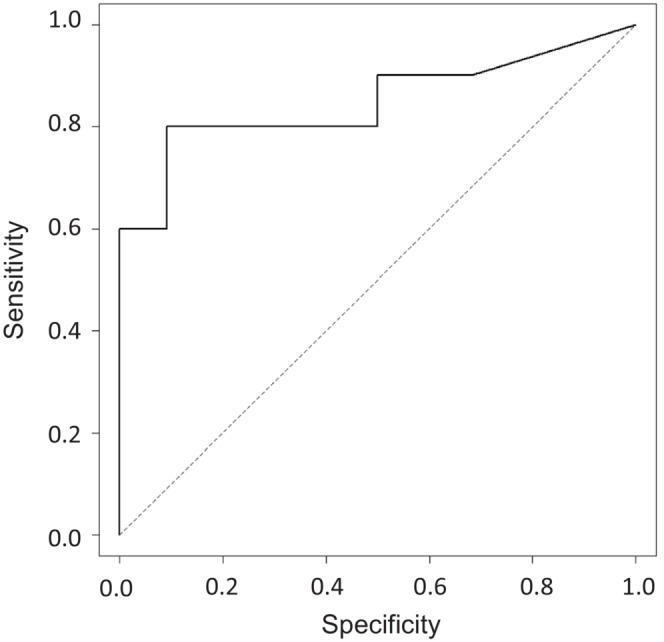
The receiver operator characteristic curve (ROC) for the halving time in the prediction of a MR^4.5^ by 48 weeks. The ROC curve showing that the cut‐off value of halving time for the prediction of a MR^4.5^ by 48 weeks was 6.9 weeks (sensitivity 0.80, specificity 0.909, ROC‐area under the curve (AUC) 0.83).

The halving time (HT) of *BCR‐ABL* ^*IS*^ was determined from the baseline and *BCR‐ABL* ^*IS*^ values using the following formula: HT = −log_10_ (2)/[(log10z−log10y)/x], where x is the number of days between the baseline and the assessment, y is the baseline *BCR‐ABL* ^*IS*^ value, and z is the assessed *BCR‐ABL* ^*IS*^ value. Shorter HTs are indicative of a more rapid decrease in *BCR‐ABL* ^*IS*^. Except for one patient whose IS% (international scale percent ratio of *BCR‐ABL* fusion transcripts to *ABL1* copies) value decreased at 8 weeks, 10 patients who achieved MR^4.5^ during this study had a shorter HT of *BCR‐ABL* ^*IS*^ % at 8 weeks (5.3 weeks; range, 3.4–44.0) compared to 22 patients without MR^4.5^ (14.1 weeks; range, 6.1–626.3), as shown by the exact Wilcoxon rank‐sum test (*p* < 0.0010). The receiver operating curve (ROC) analysis showed that the HT of *BCR‐ABL* ^*IS*^ % at 8 weeks had the best discriminatory power among variable time points that were investigated for the rate of MR^4.5^ by 48 weeks. The area under the curve (AUC) value for predicting MR^4.5^ was 0.83. The cutoff value for the predicted MR^4.5^ at 48 weeks was 6.9 weeks with a sensitivity of 80% and a specificity of 90.9% (Figure 3).

**FIGURE 4:**
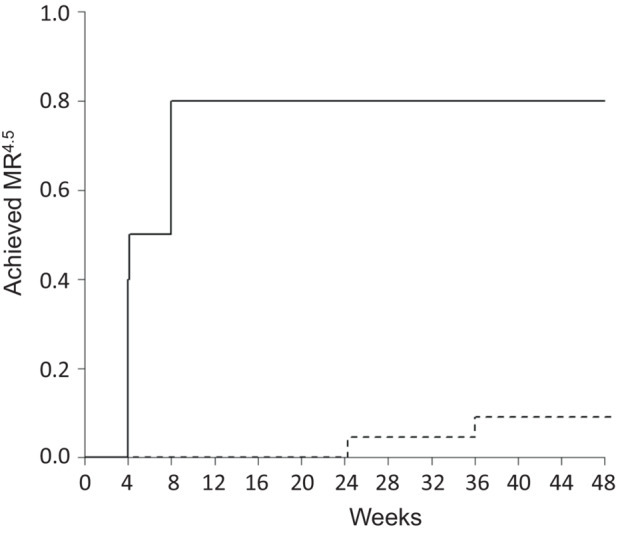
Cumulative incidences of MR^4.5^ conversion by 48 weeks according to the halving time at 8 weeks. Eight patients (80%) in the shorter halving time group achieved a MR^4.5^ during 48 weeks. The median time to MR^4.5^ was 6.07 weeks (95% confidence interval [CI], 4.00–8.00). Two patients (9%) in the longer halving time group achieved a MR^4.5^ during the 48‐week study period. The median time to a MR^4.5^ was not reached. Log‐rank test: *p* < 0.0001.

Cumulative incidences of MR^4.5^conversion by 48 weeks according to the cutoff value of HT at 8 weeks are shown in Figure 4. Eight patients (80%) in the shorter HT group achieved MR^4.5^ during 48 weeks. The median time to MR^4.5^ was 6.07 weeks [95% CI, 4.00–8.00]. Two patients (9%) in the longer HT group achieved MR^4.5^ during the 48‐week study period. The median time to MR^4.5^ was not reached. The MR^4.5^ cumulative incidence was significantly increased in the shorter HT group compared to that in the longer HT group based on the log‐rank test (*p* < 0.0001).

### 3.6 Long‐term follow‐up

Eleven patients who achieved MR^4.5^ were followed up after this study (median follow‐up: 266 days; range: 146–357 days). All patients continued the same TKI treatment but stopped TM5614 treatment after the study. Molecular responses were assessed regularly by IS‐PCR every 3 months after this study in all patients. No patient died from any cause or progressed to an advanced phase of CML. Despite discontinuing TM5614, all patients maintained MR^4.5^, except for one patient who had a loss of MR^4.5^ within 3 months after study initiation and showed fluctuations in *BCR‐ABL* ^*IS*^ values in MR^4.0^.

### 3.7 Analysis of the clinical characteristics of patients

The MR^4.5^ rate was analyzed based on the clinical characteristics shown in Table 2. Patients who were treated with TKI for 36–60 months and showed MR^4.0^ (*BCR‐ABL* ^*IS*^ value <0.01%) before the study were more likely to achieve MR^4.5^ than other patients.

**TABLE 2:** Analysis of the clinical characteristics of patients

Patients (*n* = 33)	MR^4.5^	No MR^4.5^	*p*‐value
Male	6	13	0.8033
Female	5	9	
Time since diagnosis (1)			
<36 months	1	6	0.4393
36 months – <60 months	5	5	
60 months – <120 months	3	5	
120 months	2	6	
Time since diagnosis (2)			
36 months – <60 months	5	5	0.1805
Others	6	17	
IM	1	6	0.2284
2G‐TKI	10	16	
MMR, *BCR‐ABL* ^*IS*^ value ≥0.01	1	16	0.0006
MR^4^, *BCR‐ABL* ^*IS*^ value <0.01	10	6	
TM5614 dose			
150 mg/day	6	9	0.4583
180 mg/day	5	13	

Abbreviations: 2G, second‐generation; IM, imatinib; TKI, tyrosine kinase inhibitor.

Additionally, the multivariate analyses revealed that the baseline *BCR‐ABL* ^*IS*^ value was the only factor that could significantly predict MR^4.5^ in this study (odds ratio for MR^4.5^, 55.969; 95% CI, 3.808–822.639; *p* = 0.0033; Table 3). Other factors showed no significant or marginally significant associations.

**TABLE 3:** Logistic regression analysis based on clinical characteristics

Clinical characteristics	MR^4.5^ by 42 weeks		Backward selection (*p* < 0.15)	
	OR (95% CI)	*p*‐value	OR (95% CI)	*p*‐value
Age, years	1.076 (0.980–1.181)	0.1241	1.051 (0.983–1.124)	0.1476
Sex (female/male)	0.950 (0.094–9.597)	0.9656		
TKI, 2G‐TKI/IM	25.769 (0.398–999.999)	0.1267		
Time since diagnosis, 36 months~<60 months/others	0.241 (0.009–6.579)	0.3993		
*BCR‐ABL* ^*IS*^ baseline, MR^4^ (<0.01)/MMR (≥0.01)	195.5 (2.979–999.999)	0.0135	55.969 (3.808–822.639)	0.0033
TM5614 dose, 150/180 mg/day	0.269 (0.018–4.124)	0.3456		

Abbreviations: 2G, second‐generation; CI, confidence interval; IM, imatinib; OR, odds ratio; TKI, tyrosine kinase inhibitor.

### 3.8 Safety

In 25 patients, 59 cases of any‐grade adverse events (AEs) were disclosed. The most frequent AEs were upper respiratory infections (six cases), enteritis (two cases), nasopharyngitis (two cases), zinc deficiency (two cases), limb pain (two cases), and spinal disc herniation (two cases). Three cases of severe adverse events (SAEs) were reported in two patients, including cellulitis, abdominal wall abscess, and acute hepatitis. However, we believe that these events were not related to TM5614, because they were improved by supportive care and TM5614 was continued. Although there were eight cases of AEs related to TM5614 in six patients, including lymphopenia (two cases), eyelid edema (one case), conjunctival bleeding (one case), stomatitis (one case), oral hypersensitivity (one case), liver dysfunction (one case), and headache (one case), these events were not severe and did not require any treatment. Although many of these events considered to be related appear typical of TKI, the distinction between TM5614‐related and ‐unrelated AEs was made by the physician‐in‐charge. In the pilot study prior to this Phase II trial, we measured TKI plasma concentrations before and after administration of TM5614 (data not shown). There were no differences in plasma concentrations following TM5614 administration and this finding indicated that these AEs may not be attributable to a higher exposure to TKI induced by TM5614.

## 4 DISCUSSION

We previously developed a low‐molecular‐weight synthetic inhibitor of PAI‐1^18^, ^19^ and showed that it enhances hemopoietic regeneration in a preclinical study.^25^, ^26^ We hypothesized that the inhibition of intracellular PAI‐1 activity would induce the detachment of CML‐LSCs in the BM, rendering CML‐LSCs susceptible to TKI therapy. In a mouse model of CML, we demonstrated that the combined administration of TKI and a PAI‐1 inhibitor significantly enhanced CML cell killing in the BM and prolonged CML mouse model survival.^20^ These findings provided evidence of a novel therapeutic strategy based on the inhibition of PAI‐1 activity in patients with CML.

Here, we report the TM5614 Phase II clinical trial on the use of the PAI‐1 inhibitor, TM5614 combined with a TKI for patients with CML not achieving MR^4.5^ with TKI treatment alone. The transition from an MMR/MR^4^ to MR^4.5^ was observed in 11 patients, resulting in a cumulative molecular response incidence of 33.3% at 48 weeks. During follow‐up, 10 of 11 patients maintained a MR^4.5^ for an additional year compared to those taking the same TKI alone. Before study entry, 5 patients showed transiently MR^4.5^ as the best response in 1 year but it was not sustained under the TKI monotherapy (Figure S1). The slope of *BCR‐ABL* ^*IS*^ % decreased after this study in the responder group. This figure shows that TM5614 induced MR^4.5^ in some patients without MR^4.5^.

We defined DMR as MR^4.5^, which is a *BCR‐ABL1* transcript level ≤ 0.0032% or by undetectable disease in cDNA with >100,000 *ABL* transcripts. Because assay sensitivity should be defined in a standardized manner when *BCR‐ABL1* mRNA is undetectable, DMR is also defined as MR^4^, which is a *BCR‐ABL1* transcript level ≤0.01% or by undetectable disease with the ELN definition.^3^ However, recent data suggest that the TFR success rate for patients with MR^4^ but not achieving MR^4.5^ is significantly lower than that for stable MR^4.5^.^27^ Additionally, we reported that undetectable disease, which was defined as no *BCR‐ABL1* in >100,000 *ABL1* control genes using IS‐PCR at the study baseline, was found to be predictive of TFR continuation in two independent TFR trials, the JALSG STIM213 study and STAT2.^11^, ^12^ We believe that a deeper molecular response is essential to TFR. Although DMR is the milestone of TFR, which has become the new target for CML therapy, only a small number of patients can achieve it. In the ENESTnd study, in which patients with CML were followed up for more than 10 years, the cumulative 5‐year rate of MR^4.5^ was 53.3% with nilotinib 300 mg BID and 31.4% with imatinib 400 mg QD.^22^ Among evaluable patients with no MR^4.5^ at 5 years, 38.0% (35/92) in the nilotinib arm and 22.7% (22/97) in the imatinib arm achieved an MR^4.5^ at 10 years while continuing the same treatment.^22^ Although the cumulative incidence of MR^4.5^ increased within the duration of TKI therapy, it was only 4.5%–7.6% per year. In our study, the cumulative MR^4.5^ incidence was much higher than that of the historical control, suggesting that TM5614 combined with TKI induces MR^4.5^ and TFR in patients with CML.

Patients with MR^4.5^ on TKI are a very sensitive population for whom combination therapies may pose an additional risk of negative health outcomes. During this study, bleeding events and abnormal coagulation related to the drug were not found, and TM5614 was highly safe. Therefore, the daily dose was elevated from 150 mg to 180 mg. The plasma concentrations of TM5614 were relatively stable, and no accumulation of TM5614 was observed during this study. However, late AEs related to TM5614 were reported in the preclinical study (data not shown).

In addition, in the multivariate analysis, the cumulative incidence of MR^4.5^ by 48 weeks, which was our primary objective, was associated with the molecular level, which means assessing whether an MR^4^ was achieved or not before the study. Patients with the MR^4^ achieved a significantly higher MR^4.5^ than those without MR^4^. Moreover, patients with a shorter HT (less than 6.9 weeks) of *BCR‐ABL* ^*IS*^ evaluated at 8 weeks were more likely to achieve MR^4.5^ by 48 weeks than those with a longer HT. Although the duration of TKI treatment was not related to MR^4.5^ in this study, our results show that a treatment duration of 3 years was necessary to achieve MR^4.5^, indicating that MR^4.5^ can be induced in patients treated with TKIs for at least 3 years through a combination of TKIs and TM5614. Therefore, TM5614 in combination with TKIs might be a good option for patients whose HT cannot be calculated because of *BCR‐ABL1* ^*IS*^ fluctuations at low levels around MR^4^ for more than 1 year. Such patients could not achieve MR^4.5^with traditional TKI monotherapy even if the treatment was continued for additional years.

Previous studies have shown that other approaches such as IFN‐A/peg‐IFN‐A,^28^, ^29^ a BCL‐2 inhibitor (venetoclax), a JAK2 inhibitor (ruxolitinib), a BTK inhibitor, a PPAR‐γ (pioglitazone),^30^ and a PD‐1 antibody in combination with TKI, can be applied to eliminate CML stem cells in patients with CML not achieving DMR with TKI monotherapy.^31^, ^32^ Compared to results of several ongoing randomized trials on combination drugs, including NCT02011945, NCT03610971, NCT03654768, and NCT02689440, TM5614 is relatively tolerable, which makes it a strong candidate for combinations with TKIs to eliminate CML‐LSCs.

In conclusion, TM5614 in combination with TKIs was well tolerated and induced a MR^4.5^ in more patients than that with TKI treatment alone. This combination therapy might increase the number of candidates for TFR among patients with CML compared to that with TKI monotherapy. This study has limitations. It included a small number of patients and used a comparison with historical controls. We are currently planning a phase III randomized control trial for CML‐CP patients without MR^4.5^ treated with TKIs combined with or without TM5614.

## AUTHOR CONTRIBUTIONS

**Naoto Takahashi:** Conceptualization (equal); investigation (equal); methodology (equal); project administration (equal); visualization (equal); writing – original draft (lead); writing – review and editing (lead). **Yoshihiro Kameoka:** Data curation (equal); investigation (equal); project administration (equal). **Makoto Onizuka:** Investigation (equal); methodology (equal); project administration (equal). **Yasushi Onishi:** Investigation (equal); methodology (equal); project administration (equal). **Fumiaki Takahashi:** Data curation (lead); formal analysis (lead); software (lead); supervision (lead); validation (lead); visualization (lead). **Takashi Dan:** Conceptualization (equal); data curation (equal); formal analysis (equal); funding acquisition (equal); resources (equal); software (equal); supervision (equal); validation (equal). **Toshio Miyata:** Conceptualization (lead); funding acquisition (lead); resources (lead); supervision (lead). **Kiyoshi Ando:** Conceptualization (equal); data curation (equal); formal analysis (equal); funding acquisition (equal); investigation (equal); methodology (equal); project administration (equal); supervision (equal); validation (equal); visualization (equal); writing – original draft (equal); writing – review and editing (equal). **Hideo Harigae:** Conceptualization (equal); data curation (equal); funding acquisition (lead); investigation (lead); methodology (lead); project administration (lead); writing – original draft (equal); writing – review and editing (equal).

## FUNDING INFORMATION

This study was supported by the Practical Research for Innovative Cancer Control from the Japan Agency for Medical Research and Development (AMED 19ck0106418h0002, AMED 20ck0106418h0003).

## CONFLICT OF INTEREST

N.T. declares research funding, honoraria, and speakers' bureau from Novartis, Otsuka, and Pfizer, research funding from Astellas, and honoraria from Asahikasei, Ono, Kyowa Kirin, and Mochida, outside the submitted work. Y.O. declares the speakers' bureau from Novartis and Pfizer. K.A. declares research funding and honoraria from Chugai, research funding from Novartis, Otsuka Jansen, Astellas, IQVIA, Zenyaku Kogyo, Solasia, and honoraria from Takeda, Mochida, Meiji Seika Pharma, and Kyowa Kirin. T.M. declares research funding from Astellas, Daiichi Sankyo, Kowa, Renascience, and Tokyo marine & nichido. H.H. declares research funding and speakers' bureau from Novartis, speakers' bureau and honoraria from Ono, speakers' bureau from BMS, Chugai, and Jansen, and honoraria from Astellas, and Kyowa Kirin. The other authors declare no conflict of interest.

## ETHICAL APPROVAL STATEMENT

This study was conducted in accordance with the Declaration of Helsinki and Good Clinical Practice and was approved by the Ethics Committee of Tohoku University (No. 2019–193,006) and by the ethics committees of all participating institutions.

## PATIENT CONSENT STATEMENT

All patients provided written informed consent before enrollment.

## CLINICAL TRIAL REGISTRATION

This study is a prospective, multicenter, open‐label, single‐arm phase II trial (jRCT2031190071).

## Supporting information


Figure S1

## Data Availability

The data that support the findings of this study are available from the corresponding author, NT, upon reasonable request.
