# Supplementary material for: Deep molecular response in patients with chronic phase chronic myeloid leukemia treated with the plasminogen activator inhibitor‐1 inhibitor TM5614 combined with a tyrosine kinase inhibitor
Source: Cancer Med. 2022 Sep 23;12(4):4250–8. doi: 10.1002/cam4.5292 (PMC9972105; doi:10.1002/cam4.5292)

**Supporting Information**

Supplemental figure 1: *BCR-ABL^IS^* % of each patient before and after this study. Blue circles indicate responders who achieved MR^4.5^ or a 0.5-log reduction from baseline in this study. Green circles indicate non-responders who did not achieve MR^4.5^ or a 0.5-log reduction in this study. Before study entry, 5 patients showed transiently MR^4.5^ as the best response in one year, but it was not sustained under the TKI monotherapy. The slope of *BCR-ABL^IS^* % decreased after this study in the responder group (blue circles). The halving time of *BCR-ABL^IS^* % was reduced from 527 to 404 days in this group.


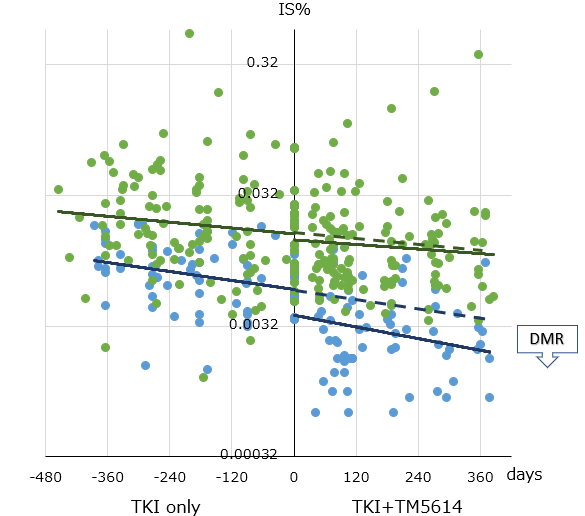

Supplement: Supplementary file 1 — Figure S1 [file CAM4-12-4250-s001.docx]
